# Supplementary material for: Seroprevalence of anti-SARS-CoV-2 IgG antibodies in the staff of a public school system in the midwestern United States
Source: PLoS One. 2021 Jun 10;16(6):e0243676. doi: 10.1371/journal.pone.0243676 (PMC8191884; doi:10.1371/journal.pone.0243676)
Supplement: S1 Fig — (DOCX) [file pone.0243676.s002.docx]

**S1 Fig:** Construction of a Causal Diagram to Identify Confounders of the Relationship Between Mask Wearing and Seropositivity

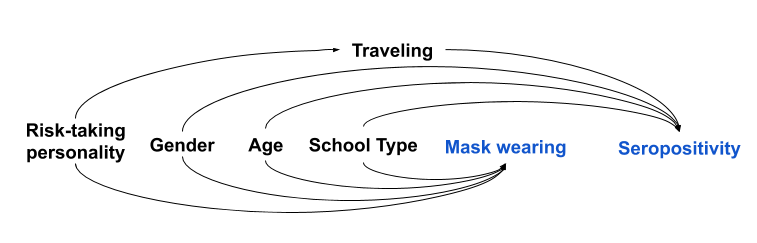


* This causal diagram is drawn under the null hypothesis of no effect of mask-wearing on seropositivity and shows the relationships between important variables hypothesized to affect both mask-wearing and seropositivity. We adjusted for these potential confounders to identify the effect of mask-wearing on seropositivity. To interpret this estimate causally, we assume that there are no unmeasured confounding variables and that our logistic model is correctly specified; however, it is possible that risk-taking personality may affect seroprevalence in other ways such as not social distancing, but those data weren’t collected and could not be adjusted for in this round of testing.”
