# Supplementary material for: Seroprevalence of anti-SARS-CoV-2 IgG antibodies in the staff of a public school system in the midwestern United States
Source: PLoS One. 2021 Jun 10;16(6):e0243676. doi: 10.1371/journal.pone.0243676 (PMC8191884; doi:10.1371/journal.pone.0243676)
Supplement: S1 Table — (DOCX) [file pone.0243676.s003.docx]

**S1 Table**: Logistic Regression Results for the relationship between mask wearing history and seropositivity, adjusting for potential confounders; Missing Data Excluded

| Effect | Relative Risk Ratio | 95% CI | | *p* |
| --- | --- | --- | --- | --- |
|  |  | *LL* | *UL* |  |
| Constant | 0.046 | 0.0037 | 0.33 | 0.0060 |
| Working in the Summer | 0.23 | 0.013 | 1.2 | 0.16 |
| Travel History | 0.42 | 0.064 | 1.5 | 0.25 |
| Working in a Middle School | 2.0 | 0.45 | 14 | 0.40 |
| Age | 1.3 | 0.49 | 3.7 | 0.56 |
| Mask History | 0.63 | 0.16 | 4.3 | 0.57 |
| Female Gender | 0.80 | 0.23 | 3.7 | 0.74 |
| Working in a High School | 0.85 | 0.13 | 6.7 | 0.86 |
| Working in Elementary School | 0.86 | 0.17 | 6.3 | 0.86 |
|  |  |  |  |  |
|  |  |  |  |  |
|  |  |  |  |  |
