# Supplementary material for: Seroprevalence of anti-SARS-CoV-2 IgG antibodies in the staff of a public school system in the midwestern United States
Source: PLoS One. 2021 Jun 10;16(6):e0243676. doi: 10.1371/journal.pone.0243676 (PMC8191884; doi:10.1371/journal.pone.0243676)
Supplement: S2 Table — (DOCX) [file pone.0243676.s004.docx]

**S2 Table**: Logistic Regression Results for the relationship between mask wearing history and seropositivity, adjusting for potential confounders; Missing Data Replaced

| Effect | Relative Risk Ratio | 95% CI | | *p* |
| --- | --- | --- | --- | --- |
|  |  | *LL* | *UL* |  |
| Constant | 0.028 | 0.0015 | 0.51 | 0.016 |
| Working in the Summer | 0.16 | 0.022 | 1.3 | 0.082 |
| Working in a Middle School | 3.4 | 0.69 | 17 | 0.13 |
| Female Gender | 0.56 | 0.19 | 1.6 | 0.29 |
| Travel History | 0.66 | 0.22 | 2.0 | 0.47 |
| Working in a High School | 1.7 | 0.31 | 9.1 | 0.55 |
| Age | 1.0 | 0.97 | 1.0 | 0.67 |
| Working in Elementary School | 1.4 | 0.26 | 7.5 | 0.69 |
| Mask History | 0.83 | 0.18 | 3.8 | 0.82 |
