# Supplementary material for: Seroprevalence of anti-SARS-CoV-2 IgG antibodies in the staff of a public school system in the midwestern United States
Source: PLoS One. 2021 Jun 10;16(6):e0243676. doi: 10.1371/journal.pone.0243676 (PMC8191884; doi:10.1371/journal.pone.0243676)
Supplement: S3 Table — (DOCX) [file pone.0243676.s005.docx]

**S3 Table:** Stepwise Backwards Feature Elimination Regression Results (Missing Data Excluded)

| Effect | Odds Ratio | 95% CI | | *p* |
| --- | --- | --- | --- | --- |
|  |  | *LL* | *UL* |  |
| Intercept | 0.028 | 0.0063 | 0.13 | <0.001 |
| Contact History | 5.6 | 2.1 | 15.1 | 0.001 |
| Previous COVID Test | 48 | 3.9 | 597 | 0.003 |
| Mask History | 0.61 | 0.13 | 2.8 | 0.53 |
| Travel History | 0.66 | 0.21 | 2.1 | 0.48 |
| Symptom History | 1.6 | 0.61 | 4.4 | 0.33 |
