# Supplementary material for: Seroprevalence of anti-SARS-CoV-2 IgG antibodies in the staff of a public school system in the midwestern United States
Source: PLoS One. 2021 Jun 10;16(6):e0243676. doi: 10.1371/journal.pone.0243676 (PMC8191884; doi:10.1371/journal.pone.0243676)
Supplement: S4 Table — (DOCX) [file pone.0243676.s006.docx]

**S4 Table:** Stepwise Backwards Feature Elimination Regression Results (Missing Data Replaced)

| Effect | Odds Ratio | 95% CI | | *p* |
| --- | --- | --- | --- | --- |
|  |  | *LL* | *UL* |  |
| Intercept | 0.027 | 0.004 | 0.097 | <0.001 |
| Previous COVID Test | 54. | 5.2 | 1300 | 0.0021 |
| Contact History | 5.1 | 1.6 | 14. | 0.0027 |
| Symptom History | 2.0 | 0.71 | 5.5 | 0.17 |
| Travel History | 0.49 | 0.11 | 1.6 | 0.29 |
| Mask History | 0.60 | 0.16 | 4.0 | 0.51 |
|  |  |  |  |  |
