# Supplementary material for: Seroprevalence of anti-SARS-CoV-2 IgG antibodies in the staff of a public school system in the midwestern United States
Source: PLoS One. 2021 Jun 10;16(6):e0243676. doi: 10.1371/journal.pone.0243676 (PMC8191884; doi:10.1371/journal.pone.0243676)
Supplement: S5 Table — (DOCX) [file pone.0243676.s007.docx]

**S5 Table:** **Stepwise Backwards Feature Elimination Regression Results, Previously COVID+ Patients Excluded (Missing Data Replaced)**

| Effect | Odds Ratio | 95% CI | | *p* |
| --- | --- | --- | --- | --- |
|  |  | *LL* | *UL* |  |
| Intercept | 0·029 | 0·0044 | 0·10 | <0·001 |
| Contact History | 6.5 | 2.06 | 18.9 | <0·001 |
| Mask History | 0·58 | 0·15 | 3.87 | 0·49 |
| Travel History | 0·36 | 0·05 | 1.33 | 0·18 |
| Symptom History | 1.62 | 0·52 | 4.57 | 0·38 |

*Note*: CI = confidence interval; *LL* = CI lower limit; *UL* = CI upper limit.
