# Supplementary material for: Seroprevalence of anti-SARS-CoV-2 IgG antibodies in the staff of a public school system in the midwestern United States
Source: PLoS One. 2021 Jun 10;16(6):e0243676. doi: 10.1371/journal.pone.0243676 (PMC8191884; doi:10.1371/journal.pone.0243676)
Supplement: S1 Text — (DOCX) [file pone.0243676.s008.docx]

**S1 Text**

Data Collection for Seroprevalence Study of COVID 19 in Lake Central Staff

**This is a student-led study with the purpose of determining the number of Lake Central staff members who have been exposed to COVID 19. If you do not wish to participate in this study, your action on this form is not necessary.**

Print: ___________________

1. Date of Birth (MM/DD/YYYY): _ _ / _ _ / _ _ _ _
2. Height (ft): _____
3. Weight (lb): _____
4. Gender: Male ❑ Female ❑ Other ❑ Prefer not to answer ❑
   1. Have you shown any of the following symptoms (check all that apply):

- Headache ❑
- Cough ❑
- Congestion ❑
- Nausea ❑
- Loss of taste/smell ❑
- Fever or Chills ❑
- Fatigue ❑
- Shortness of breath/ difficulty breathing ❑
- Muscle/body aches ❑
- Diarrhea ❑
- Sore throat ❑
  1. If yes, when did you last experience those symptoms (MM/DD): _ _ / _ _

1. 1. Have you been in contact with anyone who has been diagnosed with COVID-19: Yes ❑ No ❑
   2. If yes, when (MM/DD): _ _ / _ _
2. 1. Have you travelled outside the state in the past two weeks: Yes ❑ No ❑
   2. If yes, to where (City, State): _______________________
3. 1. Have you tested positive for COVID-19: Yes ❑ No ❑
   2. If yes, when did you test positive (MM/DD): _ _ / _ _
4. Do you wear a mask while out in public: Yes ❑ No ❑
5. If you are not a Lake Central Staff Member
   1. What is your relation to the staff member:_________________________
   2. What is the name of the staff member: ________________________

**If you are not employed by Lake Central School Corporation, please refrain from answering questions 10-13.**

1. Role at LC (Check one):

- Janitorial/Maintenance Staff ❑
- Technology Department ❑
- Lunch Staff ❑
- Bus Driver/ Bus Aide ❑
- Guidance/Administration ❑
- West Lake Department ❑
- Art, Music, or P.E. Department ❑
- English Department ❑
- Math Department ❑
- Science Department ❑
- Social Studies Department ❑
- World Language, Business, Consumer Science, or Communications Department ❑
- General Elementary Teacher ❑

1. What school do you work at (Check all that apply):

- Lake Central High School ❑
- Clark Middle School ❑
- Kahler Middle School ❑
- Grimmer Middle School ❑
- Watson Elementary School ❑
- Homan Elementary School ❑
- Peifer Elementary School ❑
- Kolling Elementary School ❑
- Bibich Elementary School ❑
- Protsman Elementary School ❑
- Transportation Center ❑

1. Do you coach a school sport: Yes ❑ No ❑
2. Do you supervise a school club: Yes ❑ No ❑
